# Can Science Help Resolve the Controversy on the Origins of the SARS-CoV-2 Pandemic?

**DOI:** 10.1128/mBio.01948-21

**Published:** 2021-08-02

**Authors:** Arturo Casadevall, Susan R. Weiss, Michael J. Imperiale

**Affiliations:** a Department of Molecular Microbiology and Immunology, Johns Hopkins School of Public Health, Baltimore, Maryland, USA; b Department of Microbiology, Perelman School of Medicine, University of Pennsylvania, Philadelphia, Pennsylvania, USA; c Department of Microbiology and Immunology, University of Michigan, Ann Arbor, Michigan, USA

**Keywords:** COVID, SARS, SARS-CoV-2

## Abstract

The origins of the calamitous SARS-CoV-2 pandemic are now the subject of vigorous discussion and debate between two competing hypotheses for how it entered the human population: (i) direct infection from a feral source, likely a bat and possibly with an intermediate mammalian host, and (ii) a lab accident whereby bat isolates infected a researcher, who then passed it to others. Here, we ask whether the tools of science can help resolve the origins question and conclude that while such studies can provide important information, these are unlikely to provide a definitive answer. Currently available data combined with historical precedent from other outbreaks and viewed through the prism of Occam’s razor favor the feral source hypothesis, but science can provide only probabilities, not certainty.

## EDITORIAL

## BACKGROUND

The COVID-19 pandemic has refocused attention on the benefits and dangers of scientific research on pathogens with pandemic potential. The benefit was apparent in the rapid development of diagnostic tools, antibody therapies, and vaccines, none of which would have been possible without a robust scientific enterprise that was able to pivot against this threat from nature within days of the outbreak. However, the magnitude of the COVID-19 tragedy has also trained a lens on the potential dangers of this type of research. In the early days of the pandemic, there was speculation that COVID-19 was a human-caused event, possibly with an engineered virus, but the latter view has little if no credence today. The preponderance of scientific evidence at this time indicates that SARS-CoV-2 is representative of a type of coronavirus that has been circulating in feral animal populations for decades (1). However, the question of whether the COVID-19 pandemic resulted from an escape event of a naturally occurring virus from a laboratory or is a direct zoonosis whereby human infection by SARS-CoV-2 was acquired from an animal remains open and has gained new life in recent months, based on largely circumstantial evidence. Although the Wuhan Institute of Virology (WIV) has been the subject of speculation as a site of origin (2–5), we make no such insinuations and instead focus on whether science can provide a definite answer for the two competing theories for the origins of SARS-CoV-2 in the absence of other information such as lab records.

## THE LAB ACCIDENT THEORY GOES FROM CONSPIRACY THEORY TO MAINSTREAM CONCERN

The COVID-19 pandemic is an extraordinary event in recent human history. The year 2020 was one of the worst years with pandemic conditions, economic collapse, political instability, and social unrest. Extraordinary events are fertile grounds for conspiracy theories. Moreover, the political climate, particularly in the United States, has been such that conspiracies, often fed by unsubstantiated opinions disseminated on social media platforms, can easily take on lives of their own. From the earliest days of the COVID-19 pandemic, there were questions as to the origin of SARS-CoV-2 and concern that it may have arisen from a laboratory event. However, for much of the first year of the COVID-19 pandemic the scientific consensus favored a zoonotic explanation and concerns about the so-called lab escape theory were voiced mainly outside scientific circles. In an earlier essay on the potential effects of the COVID-19 pandemic for gain-of-function research, we too noted that in mid-2020 the idea of a lab origin for SARS-CoV-2 was more akin to a conspiracy theory than to an accepted consideration among scientists, mainly because the emphasis then was on the idea of a deliberately engineered virus (6). However, in 2021 the concern has shifted to a possible lapse in biosafety and there has been a greater openness to consider the lab escape hypothesis now that it has been dissociated from the engineering idea. In May 2021, a group of respected scientists penned a letter urging a thorough investigation of SARS-CoV-2 origins including consideration of the lab escape hypothesis (7). This letter brought a long-simmering concern into the light, and soon the debate on SARS-CoV-2 origins was a staple for discussion in the mainstream media. On 26 May 2021, the Biden Administration asked the intelligence community (IC) to “redouble their efforts to collect and analyze information that could bring us closer to a definitive conclusion” on the origins of SARS-CoV-2 (8). In addition to spilling over to the general media, the issue has caused much debate, sometimes heated, in the scientific community (4). The lab escape hypothesis is now a mainstream concern that has the potential for enormous societal, political, and scientific ramifications depending on how the issue develops over the next few months and years.

## POSSIBLE ORIGINS

**FIG 1:**
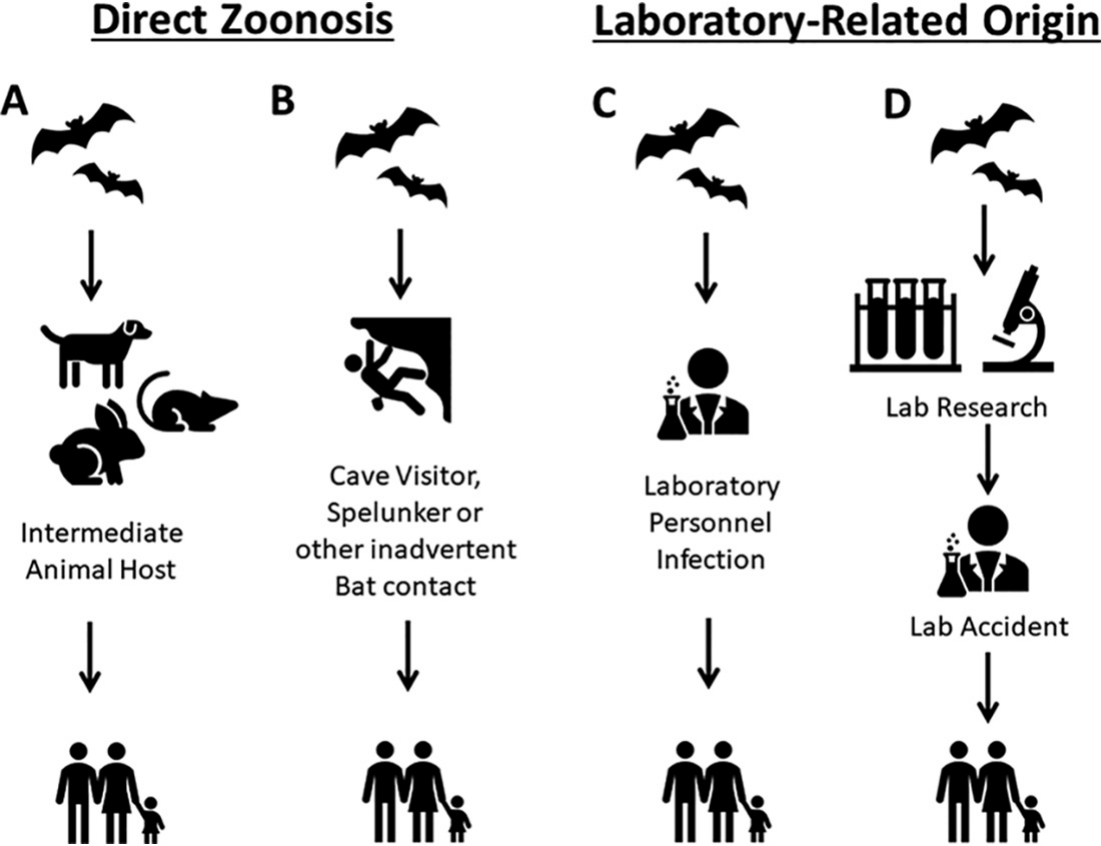
Four possible pathways for SARS-CoV-2 to enter the human population from a feral bat source. Precedents for each of the four pathways are provided in Table 1. Pathway C envisions infection of laboratory personnel involved in field work with viruses or caring for captive bats being used in research.

Based on our current knowledge of coronavirus biology and the history of infectious diseases, we can entertain four origin pathways that can be grouped into the two categories of direct zoonosis and laboratory-related origin (Fig. 1). Pathway A envisions infection of an intermediate host from a bat source with subsequent transmission and spread among the human population. Pathway B envisions direct acquisition of SARS-CoV-2 from a feral source, probably a bat, with subsequent spread among the human population. Pathway C is identical to pathway B except for intent and purpose in the sense that it occurs in an individual involved in sampling bat sources for laboratory research purposes. Pathway D involves a lab accident after SARS-CoV-2 had been isolated and brought into the laboratory, possibly adapted to growth in cell lines or laboratory animals. Pathways A and B would represent a direct zoonosis while pathways C and D would represent a laboratory-related origin of a virus that is still a zoonosis since it originally came from a feral reservoir. The literature contains precedents for the four origin pathways (Table 1).

**TABLE 1:** Precedents for the four infection pathways by which SARS-C0V-2 could have entered the human population

Pathway	Description	Precedent	Reference
Direct zoonosis			
A	Intermediate host	SARS outbreak follows transmission of SARS-CoV from bats to civets to humans	18
		Canine origin coronavirus outbreak in Malaysia	30
B	Direct infection from bat	SARS-related coronavirus (SARSr-CoV) strain RaTG13 in miners	9
Laboratory-related origin			
C	Direct infection from bat (same as pathway B except that infection occurs in course of scientific research)	As in pathway B, SARS-related coronavirus (SARSr-CoV) strain RaTG13 in miners, except that in this situation the cave visitors would be investigating bat-associated viruses	9
D	Laboratory accident	SARS infection in lab workers	14

## KNOWN KNOWNS AND UNKNOWNS

At this time, one can be almost certain that if SARS-CoV-2 emerged by lab escape (Fig. 1, pathway C or D), someone knows about it. This is because for SARS-CoV-2 to have escaped from a laboratory, scientists would have to have been working on it and people in their research group and scientific contacts would likely have discussed the work. Prior to COVID-19, there would have been no need for secrecy in this type of fundamental scientific work. To be clear, by lab escape we mean that someone became infected and carried the virus outside the laboratory in their body rather than some sort of accidental release, since the virus is not stable in air for long and even less stable on surfaces. By this explanation, COVID-19 would still be a zoonosis in the sense that SARS-CoV-2 originated from a feral animal but the trigger from the pandemic would be human activity in a research laboratory or possibly during the search in caves for bats. While such an event would not have occurred from a laboratory accident *per se*, we still lump it within the lab escape hypothesis since it would have involved an outbreak following scientific research (Fig. 1, pathway C). Of course, such an infection event could have also occurred in a cave visitor not associated with any laboratory research, such as a spelunker, in which case the origin will be a feral infection source (Fig. 1, pathway B). In fact, a respiratory disease similar to COVID-19 has been described in Chinese miners exposed to bats (9). Hence, the classification of a lab infection event would depend on the intent of the cave visitor. Therefore, we will distinguish between direct zoonosis (Fig. 1, pathways A and B), or spillover into the human population from a feral animal, and a lab accident in which a researcher was inadvertently exposed to a natural virus in a laboratory setting or in field work (Fig. 1, pathways C and D). Hence, if information was available that a direct ancestor of SARS-CoV-2 was the subject of experimentation in a laboratory prior to the human outbreak that began in 2019, that information would significantly strengthen the lab escape hypothesis (Table 2). However, such knowledge by itself would not prove that it came from a laboratory since such a virus would still have a prior animal origin and it is conceivable that a true zoonotic event still occurred that triggered the epidemic. This is in some ways similar to the investigation into the origin of the anthrax letters of 2001: although forensic evidence pointed to a particular lab, that in and of itself was not proof that USAMRIID or another laboratory was the origin (10). Hence, a lab origin event (Fig. 1, pathway D) would have required laboratory work with SARS-CoV-2, a containment breach, and infection of an initial individual or cluster of individuals who then spread the virus to others outside the laboratory. Although possible, this explanation would require the confluence of low-probability events. First, the SARS-CoV-2 or its direct feral ancestor would have to be recovered from a wild animal, probably a bat, and be brought back to the laboratory. Second, after arrival in the laboratory the virus would have to be propagated in tissue culture cells or used to infect laboratory animals, expertise that is common in virology laboratories. Indeed, serial passage through cell lines in a laboratory has been suggested as a mechanism by which a coronavirus obtained from an animal source could have acquired specificity for a human receptor (11). However, adaptation to cell lines or infection in laboratory animals would not necessarily confer human tropism, virulence, or contagiousness, attributes that are unlikely to have arisen in combination by chance alone under experimental conditions. In addition, cell culture passage can result in attenuation of a virus, as is the case for the Sabin polio vaccine. The molecular attributes responsible for human transmission are not understood, and consequently, this trait cannot be predicted or prospectively designed by genetic engineering. On the other hand, natural coronavirus isolates may have these phenotypes (12). Third, a breach of laboratory containment would have to occur resulting in human infection, an event that would require a breakdown in the physical barriers and/or laboratory safety protocols. Human infections in laboratory settings have been reported on numerous occasions, including laboratory-acquired SARS-CoV, establishing the precedent that such events can occur. Scientists working with a feral coronavirus isolate would have known about SARS and Middle East respiratory syndrome (MERS), and it can be reasonably expected that those experiments would have been conducted under appropriate biocontainment conditions to protect laboratory personnel. Laboratories that receive NIH funding must abide by U.S. biosafety regulations, although the extent of safety precautions in field work is unknown. Hence, a possible laboratory origin for SARS-CoV-2 is a known known of unknown probability. Of course, it must be acknowledged that spillover of a virus from a feral animal to the human population is also a low-probability event: an animal must be infected with a virus that is capable of infecting humans; a human must come in contact with that animal, possibly guano or its aerosols when it is contagious; the virus must replicate in the human; and the human must transmit it to others. The finding of antibodies to SARS-related coronaviruses in people who live near caves in China provides evidence for the notion of direct bat-to-human spread (13).

**TABLE 2:** Summary of evidence that would support each of the two theories

	For direct zoonosis	For laboratory-related origin
Existing evidence	Sequence similarity to other zoonotic coronaviruses	Evidence of laboratory work with coronaviruses isolated from bats
	Recent precedent for spillover of coronaviruses (SARS, MERS, canine-feline coronavirus)	Precedent of prior accidents in research laboratories
	Precedent for Spike RBD that can bind ACE2 receptor in bat and pangolin viruses	

Potential additional evidence	Discovery of SARS-CoV-2 or a direct ancestor in the wild	Evidence of laboratory work with SARS-CoV-2 or a close ancestor
	Serological evidence for SARS-CoV-2 in feral animals	Evidence that laboratory passaged a more distantly related virus that acquired the ability to bind ACE2 while retaining SARS-CoV-2 backbone
	Discovery of SARS-CoV-2 in an “intermediate” species	
	Discovery of SARS-CoV-2 in archival material	Clinical evidence that a researcher(s) was infected with SARS-CoV-2 before Dec 2019
	Finding of SARS-CoV-2 antibodies in archival human or animal serum samples	

## EVIDENCE THAT WOULD FAVOR THE LABORATORY ESCAPE HYPOTHESIS

Prior cases of laboratory infection with SARS-CoV establish that human coronavirus infections can occur in laboratory settings (14). The investigative team that the WHO sent to China to investigate the pandemic concluded that there was little evidence to support a laboratory accident (3). However, they admitted that they were not given unfettered access to the laboratory at the WIV or laboratory records. China has been criticized for not being completely transparent, but one must wonder whether any nation would readily admit to being the home to a lab accident that led to hundreds of millions of infections and almost 4 million deaths by early summer of 2021. Thus, we must ask what the best evidence is that we can expect to uncover regarding the coronaviruses being studied in Chinese laboratories. WIV is known to have been working with bat viruses (15, 16). If there is evidence that they were studying a virus whose genome is closer to that of SARS-CoV-2 than known animal isolates, that would support the argument that there was a lab escape. At this time, there is no evidence for this.

The laboratory escape hypothesis would also gain credence if deep sampling of nature reveals no close relative to SARS-CoV-2. Here, one would need to define “close” since wild coronaviruses are already 95% similar in sequence and relatives of SARS-CoV-2 are already known to be circulating in animals (1, 17). However, any negative search would have to avoid the logical problem that absence of evidence is not evidence for its absence. The natural reservoir is enormous, and it is not even clear what would define an exhaustive search with regard to sampling sites and number of samples analyzed. For SARS-CoV-1, it took more than a decade to find the bat ancestor, although the intermediate civet host was identified quite rapidly (18). For HIV, finding the natural origin took decades (19). Having said this, technological advances since SARS-CoV-1 and HIV entered the human population should accelerate these efforts. It may also be possible to rely on statistical models to get a handle on the size of the environmental reservoir. For example, if repeated sampling continues to reveal the same coronaviruses repeatedly, and discovery of new viruses reaches an asymptote, that would provide reassurance that sampling of natural sources was reaching its limits, with the caveat that nature is enormous and one could not be certain that all possible relevant ecologic niches had been sampled since the ecology of this family of viruses is not fully understood. The absence in natural isolates of certain molecular signatures that are found in SARS-CoV-2 would also support laboratory origin, although one cannot rule out the possibility that it has gone extinct in the wild since it jumped to humans.

The presence of a furin cleavage site in the Spike protein is often mentioned as evidence of laboratory manipulation. Although furin sites are unusual among sarbecoviruses and their presence has been used as an argument against direct zoonotic origin, such sites do occur in other viruses, including coronaviruses such as human CoVs HKU1 and OC43 and animal CoVs (20, 21). If no evidence of the furin cleavage sites is found in closely related environmental coronaviruses, this could strengthen an argument for cell line selection (11, 12). However, the significance of the furin cleavage site in SARS-CoV-2 may be overblown. Spike furin cleavage sites may provide optimal infectivity and/or pathogenesis for some coronaviruses but not all of them. A furin cleavage site is not necessary for the virulence of all coronaviruses, and SARS-CoV-1, a virus with approximately 10 times the mortality of SARS-CoV-2, has no such site. Interestingly, the furin site in SARS-CoV-2 is an inefficient proteolytic site relative to those found in other betacoronaviruses such as HKU1 and mouse hepatitis virus (MHV) (22). Furthermore, among MHV strains, while most have cleavage sites and encode efficiently cleaved spikes, the MHV2 strain encodes an uncleaved spike and is highly pathogenic (23) and cleavage at the S1/S2 site can be carried out by proteases other than furin (22). A better understanding of molecular motifs associated with certain feral species could provide additional clues, but that too could come about only from extensive reconnaissance of the natural reservoirs combined with experimental work on cell tropism and pathogenesis, knowledge that is currently unavailable. However, any virus adapted either to laboratory conditions or to an intermediate host could have accumulated sufficient sequence changes such that finding its ancestor in the wild may no longer be possible with certainty. Hence, short of more transparency from laboratory operations or human disclosure, deep sampling and genomic analysis alone are unlikely to provide definitive information to support the lab escape hypothesis.

A negative environmental search with enough statistical power to ensure that the sampling was sufficient, combined with establishing the relevance of a confluence of improbable coincidences, would provide a strong circumstantial case for the lab escape hypothesis. These coincidences include that the outbreak began in Wuhan, which is the only city in China with a biosafety level 4 (BSL-4) laboratory; an unverified newspaper report of laboratory members being treated for a COVID-19-like respiratory illness prior to the outbreak (24); and the rapid emergence of asymptomatic transmission, which set SARS-CoV-2 apart from the SARS and MERS coronaviruses and suggests the possibility of adaptation to an intermediate host prior to entering humans. However, these coincidences need to be critically evaluated. For example, the fact that the WIV has the only BSL-4 facility in China is not necessarily relevant when much of the work on coronaviruses is carried out under BSL-3 conditions, which could include many other laboratories. Of more concern is the acknowledgment that WIV was working with some of these viruses under BSL-2 containment (25), a biosafety level at which accidents are more likely to occur. The report of respiratory illnesses in laboratory personnel is significant only if they were caused by SARS-CoV-2. The human contagiousness of SARS-CoV-2 relative to the agents of SARS and MERS is important only if we knew that this trait was rare among feral coronaviruses, something that we currently don’t know. SARS was contagious and efficiently transferred by airborne spread as shown by an outbreak among airline passengers exposed to a single infected individual (26). Hence, a confluence of coincidences is relevant only if these can be factually tied to emergence of COVID-19.

## EVIDENCE THAT WOULD FAVOR DIRECT ZOONOSIS

If a SARS-CoV-2 close relative cannot be found in a wild animal, what evidence would be more convincing than the current genetic analyses that have been presented? To answer this question, it is first best to summarize the current state of affairs. First, SARS-CoV-2 Spike has a receptor binding domain (RBD) that is similar to that of SARS-CoV-1, and it is predicted to bind somewhat less well. In addition, similar RBDs have been found in coronaviruses isolated from pangolins (27). Second, there is the polybasic cleavage site that would allow the Spike protein to be cut by furin proteases as discussed above.

Certainly, the search for close relatives needs to be continued. If deep sampling of nature reveals a close relative of SARS-CoV-2, that would be strongly suggestive and supportive of a zoonotic origin but would not be conclusive in itself. The percentage of sequence identity to qualify as a “close relative” is currently uncertain since we have limited information on the extent of sequence variation among related feral coronaviruses, but that knowledge would emerge from the sampling and genomic sequencing efforts. However, based on the fact that current coronavirus sequences are already closely related, the required identity would have to be quite high, perhaps approaching 100%. A recent study of 411 bat samples from a small geographic region in China identified 24 coronaviruses including four close relatives of SARS-CoV-2 (28). Such diversity implies that finding the direct ancestor of SARS-CoV-2 could require a tremendous effort. However, identification of a virus almost identical to SARS-CoV-2 in an intermediate species like the civet for SARS-CoV-1 and the camel for MERS-CoV would provide further evidence of a zoonotic origin. However, even if someone prospectively finds a close relative in nature, one cannot rule out the possibility that that same virus had not been worked on in the lab and released accidentally, or that the virus was passed from a human to an animal.

A major caveat in environmental sampling studies is that humans have already passed SARS-CoV-2 to numerous other animals including zoo animals, pets, etc., and human-derived virus could find its way to the feral reservoir (anthroponosis). Finding SARS-CoV-2 in archival clinical specimens that predate fall 2019 would be powerful evidence against lab escape. This type of evidence was used to date the entrance of HIV into human populations and refute human-origin theories associated with vaccination campaigns in Africa and intentional human design. Serology on archived blood samples could also be useful with the caveat that antibody cross-reactivity between SARS-CoV-2 and endemic coronaviruses (29) would require a careful and rigorous investigation.

If human detective investigations, including the ongoing project by the U.S. intelligence community (IC), find no evidence that scientists were working on a SARS-CoV-2-like virus at the Wuhan laboratory, this would provide powerful evidence against the lab escape hypothesis. Whether or not the IC concludes that there is strong evidence for a lab exposure, it would be preferable for them to make that evidence available for scrutiny. However, sometimes disclosing such evidence is not possible because it makes known intelligence-gathering processes that need to be protected. If so, the IC should provide a classified briefing for trusted members of the scientific community who hold security clearances, such that those scientists can attest to the validity of the intelligence data without revealing the details. Again, such evidence would encounter the logical problem posed by Aristotle’s argument from ignorance: absence of evidence is not evidence for its absence. Even if no evidence for human work on SARS-CoV-2 is found, critics may argue that a cover-up was in place.

Also supporting a zoonotic origin are historical precedents for prior pandemics, including the 1918 and 2009 flu pandemics, SARS-CoV-1, MERS-CoV, and HIV. In fact, a cluster of patients being screened for SARS-CoV-2 were recently found to be infected with a novel coronavirus of canine origin in Malaysia (30), suggesting that zoonotic coronavirus events may be quite common. But once again, the problem is that each epidemic is different and history, while providing precedents suggesting that zoonosis is likely and possible, is not conclusive. In this regard, the 1977 reappearance of H1N1 virus could have been caused by vaccination experiments, providing a precedent of how even well-intentioned medical research can facilitate an epidemic (31).

## THE PROVENANCE QUESTION IN THE CONTEXT OF SCIENTIFIC THINKING

In contrast to other disciplines such as religion that operate with a knowledge base that comes from received truths, and law, where certainty beyond reasonable doubt is a legal standard, science prides itself in that it considers all scientific knowledge as provisional. Hence, this essay is written with anticipation that what we know about the provenance of SARS-CoV-2 will change as more information becomes available. At this time, it appears unlikely that a “smoking gun” supporting one or the other theory will be found any time soon. Furthermore, as is apparent from the discussion above, any scientific evidence will be conditional on a set of probabilities. Scientists are comfortable with uncertainty, but conclusions on the provenance question stated as a set of likelihoods may not be satisfying to the public and policy makers. Nonetheless, the scientific community should not automatically dismiss reasonable nonscientific speculation: we cannot presume that only science holds the answers. Conversely, the nonscientific public should become more conversant in the scientific method, to allow them to understand why it is difficult for scientists to put a lot of weight on much of the circumstantial evidence for lab escape. Also guiding scientific thought is Occam’s principle, which is a time-tested belief that the simplest explanation is likely to be the correct one and should be favored until invalidated by new evidence. Currently, the principle of parsimony or Occam’s Razor favors the simplest explanation, that COVID-19 was a direct zoonosis perhaps involving an intermediate species. However, scientists function in society, and communication between scientists and the lay public who support science is often difficult and fraught with the potential for miscommunication. This problem is succinctly stated by Maggie Bender in an article analyzing the origins of SARS-CoV-2 (32):

Science values possibility, but people value certainty. So far, science communication hasn’t been able to bridge the two successfully. And during the largest public health crisis of a generation, that disconnect has had disastrous consequences. This discomfort with probability and an overreliance on false assuredness are the issues at the heart of the debate over the origins of SARS-CoV-2.

## CONCLUSION

A summary of the evidence that would support the two theories is presented in Table 2 with the possible routes of infection illustrated in Fig. 1 and Table 1. Considering the currently available evidence, we favor the direct zoonosis hypothesis, namely, that SARS-CoV-2 most likely entered the human population from close contact between an index case, or cases, and an infected animal. However, we agree that both possibilities need further exploration. Nevertheless, the best science combined with other types of investigation may not provide a definitive answer. Even the best chance to ascertain what happened using science alone, which is deep sampling of the natural reservoirs, may not be conclusive.

In the absence of clear-cut answers, we need to continue to study these viruses in the laboratory since science is the best human defense against pandemics. Preparedness for the next pandemic requires not only robust public health systems but also a strong scientific research base as evidenced by the fact that the most effective response against COVID-19 was development of vaccines, drugs, monoclonal antibodies (MAbs), and plasma—all of which needed scientific backup dependent on knowledge of pathogenesis and immunology of coronaviruses.
